# Transcultural adaptation and validation of Persian Version of Patient Assessment of Chronic Illness Care (PACIC-5As) Questionnaire in Iranian older patients with type 2 diabetes

**DOI:** 10.1186/s12913-024-11557-0

**Published:** 2024-09-16

**Authors:** Sahar Maroufi, Leila Dehghankar, Ahad Alizadeh, Mohammad Amerzadeh, Seyedeh Ameneh Motalebi

**Affiliations:** 1 Student Research Committee, Qazvin University of Medical Sciences, Qazvin, Iran; 2 Social Determinants of Health Research Center, Research Institute for Prevention of Non-Communicable Diseases, Qazvin University of Medical Sciences, Qazvin, Iran; 3 Department of Nursing, Guilan University of Medical Sciences, Rasht, Iran; 4 Medical Microbiology Research Center, Qazvin University of Medical Sciences, Qazvin, Iran; 5 Non-communicable Diseases Research Center, Research Institute for Prevention of Non-communicable Diseases, Qazvin University of Medical Sciences, Qazvin, Iran

**Keywords:** Aged, Diabetes mellitus, Chronic disease, Health care, Psychometrics

## Abstract

**Background:**

The Patient Assessment of Chronic Illness Care (PACIC-5As) questionnaire has been designed to evaluate the healthcare experiences of individuals with chronic diseases such as diabetes. Older adults are at higher risk for diabetes and its associated complications. The aim of this study was transcultural adaptation and evaluation of the validity and reliability of the PACIC-5As questionnaire in older patients with diabetes residing in Qazvin City, Iran.

**Method:**

In this validation study, we recruited 306 older patients with diabetes from Comprehensive Health Centers in Qazvin, Iran. The multi-stage cluster sampling technique was used to choose a representative sample. The PACIC-5As questionnaire was translated into Persian using the World Health Organization (WHO) standardized method. The validity (face, content, and construct) and reliability (Cronbach’s alpha) of the PACIC-5As were assessed. Data analysis was conducted using R software and the Lavaan package.

**Results:**

The mean age of the older patients was 69.99 ± 6.94 years old. Most older participants were female (*n* = 180, 58.82%) and married (*n* = 216, 70.59%). Regarding face validity, all items of PACIC-5As had impact scores greater than 1.5. In terms of content validity, all items had a content validity ratio > 0.49 and a content validity index > 0.79. The results of confirmatory factor analysis demonstrated that the model exhibited satisfactory fit across the expected five factors, including assess, advise, agree, assist, and arrange, for the 25 items of the PACIC-5As questionnaire. The Cronbach’s alpha coefficient for the PACIC-5As questionnaire was 0.805.

**Conclusion:**

This study indicates that the Persian version of the PACIC-5As questionnaire is valid and reliable for assessing healthcare experiences in older patients with diabetes. This means that the questionnaire can be effectively used in this population.

**Supplementary Information:**

The online version contains supplementary material available at 10.1186/s12913-024-11557-0.

## Background

The global population is aging rapidly [1]. The fast growth of older people brings significant challenges, particularly relating to their health [2]. Approximately 75% of individuals aged 60 and above are affected by at least one chronic disease, with nearly 50% experiencing two or more chronic conditions [3]. Diabetes mellitus is among the most common and preventable chronic diseases [4]. The prevalence of diabetes is highest among older adults [5]. Nearly half of all individuals with diabetes are older adults (aged 65 years or older) [6]. The older adult population is one of the fastest-growing segments of the diabetes population. It is projected that these numbers will grow dramatically over the next few decades [7]. In Iran, Rashidi et al. (2017) found that approximately 14.4% of older adults have been diagnosed with type 2 diabetes [8].

Diabetes has emerged as one of the most serious and prevalent chronic diseases, posing a life-threatening risk, debilitating complications, substantial costs, and a reduction in life expectancy [9]. Older adults with diabetes are at serious risk of both micro and macrovascular complications [10]. Diabetes leads to an increased need for healthcare services, home care, hospitalization, and even residency in nursing homes [11]. Since self-care is a crucial aspect of diabetes management, patients need to adopt proper lifestyle habits and gain sufficient knowledge about the disease and its treatments [12].

Appropriate management of diabetes poses a significant challenge for individuals with the condition and healthcare providers [13]. Diabetes in older patients is a significant public health concern in the 21st century, and when combined with other health conditions, it can lead to exacerbated side effects [14]. Furthermore, older adults with diabetes require different types and qualities of care than other patient groups due to physiological, psychological, and social changes [15]. Iran is one of the most populous countries in the Middle East, where diabetes management is currently inadequate. Previous systematic reviews have shown that people aged over 55, especially women, tend to have poorer diabetes management [16]. A nationwide analysis of data for 30,202 patients revealed that only 13.2% of individuals with diabetes successfully attained satisfactory levels of glycemic control [17].

Managing diabetes in this group requires a comprehensive healthcare system that includes diagnosis, monitoring, and continuous medical treatment. The McColl Institute for Health Innovation developed the Chronic Care Model (CCM) to guide the delivery of healthcare services to patients with chronic conditions [18]. The CCM is a conceptual framework designed to bridge the gap between clinical research and real-life medical practice [19]. It focuses on providing proactive and planned care for chronic diseases rather than reactive and unplanned care. The CCM has six key dimensions: organization of health care, clinical information systems, delivery system design, decision support, self-management support, and community resources. It has been widely accepted for improving the care of chronically ill patients. Specifically, the “self-management support” aspect of the CCM helps patients enhance their confidence and skills to manage their illness better [20].

The Patients Assessment Chronic Illness Care (PACIC) questionnaire, developed by Glasgow et al., is used to assess patient care for chronic diseases based on the CCM [21]. It is a self-reporting instrument that provides patients’ perspectives on receiving care for chronic diseases [22]. While there are several tools to measure patients’ experiences of chronic care [23], PACIC is one of the most suitable instruments to measure the chronic care management experiences of patients as it assesses the level of alignment with the CCM [24, 25]. The initial version of the questionnaire comprises 20 items divided into five subscales: patient activation, decision-making support, goal setting, problem-solving, and follow-up [26]. Glasgow et al. [24] expanded the PACIC questionnaire by including six additional items to assess the recommended 5As model of chronic disease care in accordance with the guidelines of the United States Preventive Services Task Force [27]. The “5As” model is an evidence-based approach to behavior change employed to improve patients’ self-management. The primary elements of this model include assessing current behavior (assess), counseling the patient (advise), reaching a shared agreement on realistic goals (agree), assisting the patient throughout the lifestyle change (assist), and providing ongoing follow-up (arrange) [28]. The PACIC-5As model has been adapted into several languages, including Hindi [29], Danish [30], French [31], Korean [32], Thai [33], Bahasa Melayu [34], Arabic [35], German [36], and Spanish [37].

Diabetes incidence has increasingly risen in Iran, especially among the older adults. Several reports consistently highlight that diabetic patients in Iran largely do not receive the necessary quality healthcare services [38]. Due to the significant role of culture and context in perceiving diabetes care [39], it needs for a questionnaire that has acceptable validity, reliability, and cultural appropriateness for older Iranian patients with diabetes. Currently, there is a lack of suitable tools to evaluate the quality of care for chronic diseases in older Iranians. The PACIC-5 A is an invaluable tool for evaluating the structure of primary care and self-management support in diabetes from the patient’s viewpoint. Therefore, this study aimed to determine the validity and reliability of the PACIC-5As instrument in a sample of older patients with diabetes in Qazvin, Iran.

### Objectives

The objectives of this study are the following: (1) to translate and to assess the psychometric quality of the PACIC using the appropriate psychometric tests; (2) to determine the relationship between PACIC-5As scores and older patient characteristics and the quality of diabetes-specific care received.

## Methods

This validation study was carried out on 306 older patients referred to the health centers in Qazvin, Iran. The multi-stage cluster sampling method was performed for selecting the older patients. For this purpose, Qazvin City was divided into three urban zones. Then, nine Comprehensive Health Centers randomly selected from the zones (three from the first, four from the second, and two from the third zone). In each center, a certain number of older patients with diabetes were randomly identified, and they were then called for the initial screening. During the phone call, potential participants were provided with an explanation of the study’s purpose and procedures. Older patients who met the inclusion criteria were invited to come to the health centers near the region where they resided, at a certain time. The questionnaires were completed through face-to-face interviews by the first author.

Inclusion criteria were being 60 years old or over a diagnosis of diabetes for at least six months (by a specialist physician), and willingness to participate in the study. Older patients with severe physical or mental problems that hindered their ability to communicate were excluded from the study.

In Confirmatory Factor Analysis (CFA) studies, one way to determine sample size is to use the Rule of Thumb, which recommends having at least ten participants for each item in the questionnaire. It also suggests that sample sizes larger than 300 participants are generally considered suitable for most structural models in confirmatory factor analysis [38]. As a result, 306 participants were chosen for this study.

### Instruments

Data were collected using a demographic and clinical characteristics checklist and the PACIC-5As questionnaire.

The socio-demographic and clinical characteristics checklist elicited information from older patients that included age, gender, marital status, education level, occupation, economic status, living arrangement, duration of diabetes, type of diabetes treatment, and diabetes-related complications.

PACIC-5As questionnaire: The PACIC questionnaire consists of 20 items and five domains of assessment (items 1, 11, 15, 20, 21), advise (items 4, 6, 9, 19, 24), agree (items 2, 3, 7, 8, 25), assist (items 10, 12, 13, 14, 26), and arrange (items 16, 17, 18, 22, 23). The questionnaire items are rated on a 5-point Likert scale, ranging from “never” with a score of 1 to “always” with a score of 5, where a higher score indicates better patient assessment of chronic illness care [24].

### Translation process

After correspondence with the questionnaire designer and obtaining permission, the translation process was conducted according to the protocols of the World Health Organization (WHO) [40] as follows:

### Translation of the questionnaire into Persian

Two individuals who are proficient in both English and Persian independently translated the English version of the questionnaire into Persian. One of the translators had familiarity with medical sciences and their terminology. Ultimately, we obtained two independent Persian translations of the PACIC-5As.

In the second stage, the Persian translation of the questionnaire and revised by the project executors and colleagues. They took into account all options for word or phrase equivalency to prepare a unified Persian version of the questionnaire.

#### Retranslation into English

In this stage, two additional translators, fluent in both languages, translated the Persian version back into English. Then, the final English version was compared to the original questionnaire by two separate translators, and minor translation errors were found and fixed.

### Validity of the questionnaire

The face, content, and structural validity of the questionnaire were examined.

### Face validity assessment

For quantitative face validity, 10 older individuals with diabetes were asked to rate the importance of each item in the questionnaire on a 5-point Likert scale, ranging from 1 (not important at all) to 5 (very important). The impact scores were calculated using the following formula:

$$Item\,Impact=Frequency(\%)\times Importance\,Score$$

The term “frequency” refers to the percentage of individuals who rated the item with scores of 4 or 5, while “importance” represents the average importance rating based on the Likert scale.

The item impact score should not be less than 1.5 to accept the face validity of each item. Only questions with item impact scores higher than 1.5 are considered acceptable in terms of face validity [41]. The impact scores of all items of PACIC-5As were greater than 1.5 (Table 1).

**Table 1:** The I-CVI and CVR for the PACIC-5As scale items

Items	Item Impact	I-CVI	CVR
		Relevancy	Essential
1. Asked for my ideas when we made a treatment plan	4.6	1.00	1.00
2. Given choices about treatment to think about	4.2	0.87	0.60
3. Asked to talk about any problems with my medicines or their effects	4.8	1.00	1.00
4. Satisfied that my care was well organized	5.0	1.00	0.73
6. Shown how what I did to take care of my illness influenced my condition	4.1	1.00	0.87
7. Asked to talk about my goals in caring for my illness	4.2	0.93	0.87
8. Helped to set specific goals to improve my eating or exercise	5.0	1.00	0.73
9. Given a copy of my treatment plan	4.5	1.00	1.00
10. Encouraged to go to a specific group or class to help me cope with my chronic illness	3.8	0.93	0.73
11. Asked questions, either directly or on a survey, about my health habits	4.4	1.00	0.87
12. Was sure that my doctor or nurse thought about my values and my traditions when they recommended treatments to me	3.5	0.93	0.87
13. Helped to make a treatment plan that I could carry out in my daily life	4.9	1.00	1.00
14. Helped to plan ahead so I could take care of my illness, even in hard times	5.0	1.00	0.87
15. Asked how my chronic illness affected my life	4.0	0.93	0.87
16. Contacted after a visit to see how things were going	4.4	0.87	0.60
17. Encouraged to attend programs in the community that could help me	4.4	0.93	0.73
18. Referred to a dietitian, health educator or counsellor	5.0	1.00	1.00
19. Told how my visits with other types of doctors, an eye doctor or surgeon, helped my treatment	5.0	0.87	0.73
20. Asked how my visits with other doctors were going	4.7	0.87	0.73
21. Asked what I would like to discuss about my illness at that visit	2.4	0.93	0.60
22. Asked how my work, family or social situation related to taking care of my illness	3.1	1.00	0.73
23. Helped to make plans for how to get support from my friends, family or the community	2.9	0.93	1.00
24. Told how important the things I do to take care of my illness (e.g. exercise) were for my health	5.0	1.00	0.73
25. Set a goal together with my team for what I could do to manage my condition	4.9	1.00	0.87
26. Given a book or monitoring log in which to record the progress I make-	2.1	1.00	1.00

*I-CVI* Items Content Validity Index, *CVR* Content Validity Ratio

#### Content validity assessment

Both qualitative and quantitative methods were used to determine the validity of the content.

For qualitative assessment, the questionnaire was provided to 15 experts in geriatric medicine, geriatric nursing, nursing education, and endocrinology. They were asked to review the questionnaire based on criteria such as adherence to grammar rules, using appropriate terminology and proper phrases, and providing necessary feedback.

In the quantitative method for determining content validity, the content validity ratio (CVR) and content validity index (CVI) were calculated.

To determine the CVR, experts were initially requested to review each item based on a 3-point Likert scale (1. not essential, 2. useful but not essential, 3. essential). Then, the CVR was calculated using the following formula:

$$CVR=(ne-N/2)/(N/2)$$

In the above formula, “ne” represents the number of experts who rated the item as “essential,” and “N” represents the total number of experts [42].

If the resulting value from the formula is greater than the critical value from the Lawshe table (which is 0.49, determined based on the evaluation of 15 experts), it indicates that the presence of the corresponding item is statistically significant (*p* < 0.05) and necessary in this instrument [43].

The CVI was used to determine the relevance of the questionnaire items. The questionnaire was given to 15 experts, who rated each statement on a 4-point Likert scale (1. not relevant at all, 2. somewhat relevant, 3. moderately relevant, 4. completely relevant). The CVI score was then calculated by adding up the scores for each item rated 3 or 4, and dividing by the total number of respondents. According to the guidelines, items with scores above 0.79 were kept in the questionnaire. Items with scores between 0.70 and 0.79 were considered borderline and needed to be revised, and items scoring less than 0.70 were considered unacceptable and should be removed [44]. The CVR and I-CVI for the items of PACIC-5As are presented in Table 1.

### Construct validity assessment

#### Structural validity assessment

To determine the number of factors in the scale, CFA was performed using common goodness-of-fit indices in Structural Equation Modeling (SEM) with R software and the Lavaan package.

### Reliability

To assess the reliability and internal consistency of the questionnaire, Cronbach’s alpha coefficient was used. An internal consistency above 0.70 is considered acceptable [45].

### Ethical consideration

The study was approved by the Ethics Committee of Qazvin University of Medical Sciences, Qazvin, Iran (IR.QUMS.REC.1401.148). Participants were informed about the objectives, procedures, potential benefits, and drawbacks of the study. They were instructed that participation was voluntary and that the confidentiality of the obtained information was guaranteed. Informed consent was obtained from all respondents.

### Data analysis

R software version 4.2.2 and the Lavaan package were used for data analysis. Descriptive statistics such as mean and standard deviation were used to describe quantitative variables, including age, duration of diabetes, and PACIC-5As scores. Frequency and percentage were utilized to describe qualitative variables, including gender, marital status, education level, occupation, economic status, living arrangement, type of diabetes treatment, and diabetes-related complications. In the CFA model, the most common goodness-of-fit indices were used to assess the fit of the proposed model based on an acceptable threshold obtained through maximum likelihood estimation. The reliability and internal consistency of the questionnaire were assessed using Cronbach’s alpha coefficient. The multivariate regression model was used to determine the predictors of older patients’ perspective of diabetic care. Normal distribution of the data was confirmed by the skewness (0.078) and the kurtosis (4.329) values that were in the acceptable ranges. As Byrne (2010) [46] stated that if the skewness value is between − 2 to + 2 and the kurtosis value is between − 7 to + 7, normality of the data could be assumed. The multicollinearity issues was assessed by the variance inflation factor (VIF) and none of variables had VIF more than 5. The homoscedasticity was also evaluated and confirmed. The statistical significance level was set at *p* < 0.05.

## Results

The mean age of the participants was 69.99 ± 6.94 years, ranging from 60 to 96 years. The average age of diabetes onset was 12.46 ± 5.9 years. Most older patients (*n* = 230, 75.16%) were utilizing insulin injections as part of their treatment regimen. The demographic and clinical characteristics of the older patients are presented in Table 2.

**Table 2:** The demographic and clinical characteristics of the population

Age (years), (mean ± SD)	69.99 ± 6.94
Gender, n (%)	
Female	180 (58.82)
Male	126 (41.18)
Marital Status, n (%)	
Married1	216 (70.59)
Single4	3 (0.98)
Divorced2	7 (2.29)
Widowed3+	80 (26.14)
Educational level, n (%)	
Illıtrate	65 (21.24)
Elementary	167 (54.58)
High school and diploma	52 (16.99)
Academic	22 (7.19)
Job, n (%)	
Retired	137 (44.77)
Housewife	121 (39.54)
Employed	25 (8.17)
Unemployed	23 (7.67)
Living arrangement, n (%)	
With spouse	138 (45.10)
With children	46 (15.03)
With spouse and children	74 (24.18)
Other	8 (2.61)
Alone	40 (13.07)
Economic status, n (%)	
Low	10 (3.27)
Average	92 (30.07)
Good	186 (60.78)
Excellent	18 (5.88)
Treatment type	
Oral	76 (24.84)
Insulin injection	209(68.30)
Combination therapy	21(6.86)
Long-term side effects	
Yes	210(68.63)
No	96(31.37)

Based on the information in Table 3, the mean score of PACIC-5 A was 3.47 ± 0.24. The highest level was in advice (4.13 ± 0.30) and the lowest level was in arrange (3.21 ± 0.30).

**Table 3:** Descriptive statistics of PACIC 5 A and its domains (*n* = 306)

Subscales	Mean	SD	Median	IQR	Min	Max
Agree	3.32	0.36	3.20	0.60	2.20	5.00
Assist	3.26	0.30	3.20	0.40	2.20	4.20
Advice	4.13	0.30	4.20	0.40	3.00	5.00
Arrange	3.21	0.30	3.20	0.40	2.20	4.60
Assess	3.42	0.40	3.40	0.40	1.80	5.00
Overall	3.47	0.24	3.48	0.28	2.40	4.60

*SD* Standard Deviation, *IQR* Interquartile Range

The multivariate regression model showed that level of education was the significant predictor of older patients’ perspective of diabetic care; older patients had a better perspective if they had secondary and diploma (β = 0.11, *p* = 0.003) or academic (β: 0.23, *p* **< 0**.001) educational level (Table 4).

**Table 4:** Predictors for older patients’ perspective of diabetes care

Variables		Beta (CI: 95%)	*P*
Age		-0.12(-0.01,0.00)	0.071
Gender	Female	**-**0.01(-0.08,0.07)	0.965
Marital status	Married	1	1
	Single	-0.01(-0.33,0.27)	0.836
	Divorced	-0.07(-0.29,0.06)	0.185
	Widowed	0.14(-0.07,0.23)	0.312
Level of education	Illiterate	**-0.14(-0.16**,**-0.01)**	**0.020**
	Elementary	1	1
	Secondary and diploma	**0.18(0.04**,**0.19)**	**0.003**
	Academic	**0.25(0.12**,**0.34)**	**< 0.001**
Economic situation	Low	-0.01(-0.16,0.15)	0.911
	Average	-0.01(-0.07,0.23)	0.864
	Good	1	1
	Excellent	-0.02(-0.13,0.09)	0.766
Job	Unemployed	1	1
	Employed	0.01(-0.10,0.12)	0.860
	Retired	0.01(-0.07,0.08)	0.922
Living arrangement	With spouse	1	1
	With spouse and children	0.01(-0.06,0.08)	0.740
	With children	-0.14(-0.24,0.05)	0.204
	Other	-0.05(-0.27,0.13)	**0.486**
	Alone	-0.17(-0.29,0.05)	**0.157**

*Beta* Adjusted β coefficient, *CI* Confidence Interval

### CFA results

**Fig. 1:**
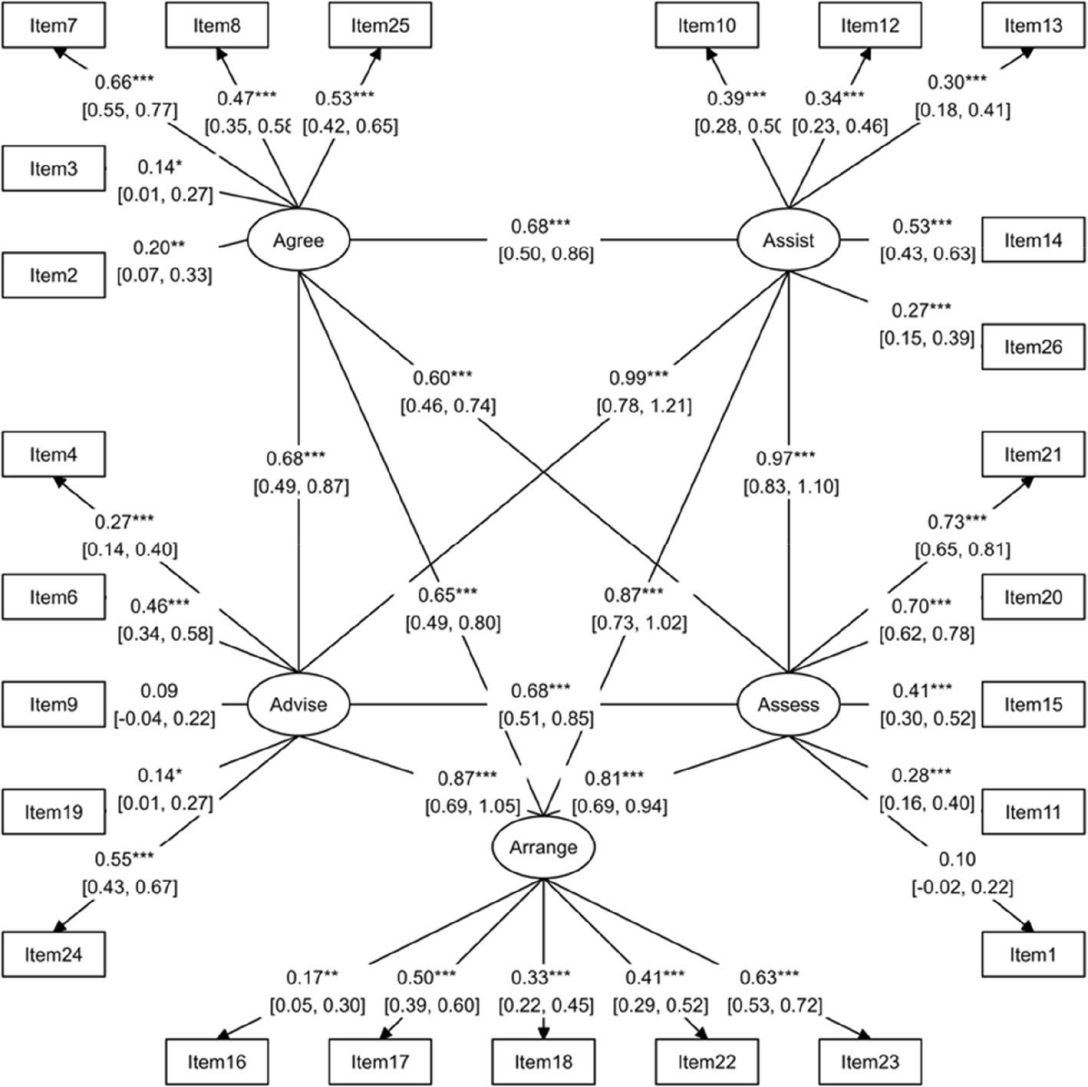
The five-factor model for PACIC-5As obtained from confirmatory factor analysis

Based on the information in Table 5, the Structural Equation Modeling (SEM) analysis of the proposed model yielded a reasonable fit, with a Root Mean Square Error of Approximation (RMSEA) of 0.07 and a chi-squared/df ratio of 2.539, both indicating acceptable model fit [47]. The Goodness-of-Fit Index (GFI) of 0.85 and Adjusted Goodness-of-Fit Index (AGFI) of 0.81 suggest a moderately good fit, while the Incremental Fit Index (IFI) of 0.72 and Comparative Fit Index (CFI) of 0.71 slightly fall below the recommended threshold of 0.90 for good fit. The Standardized Root Mean Square Residual (SRMR) of 0.08 is within the acceptable range of less than 0.08, further supporting the overall reasonable fit of the SEM model. The structural model of the questionnaire is presented in Fig. 1.

**Table 5:** Fit indices for CFA of the PACIC-5As questionnaire in older patients with diabetes

Fit Index	Abbreviated Indicator	Values
Chi-Square/DF	*χ*^2^/DF	2.539
Root Mean Square Error of Approximation	RMSEA	0.071
Goodness of Fit	GFI	0.85
Adjusted Goodness of Fit Index	AGFI	0.81
Incremental Fit Index	IFI	0.72
Comparative Fit Index	CFI	0.71
Standardized Root Mean Square Residual	SRMR	0.08

## Discussion

This study aimed to evaluate the psychometric properties of the Persian version of the PACIC-5As questionnaire in older adults with diabetes. The study also sought to establish the correlation between PACIC-5As scores and the characteristics of older patients, as well as the quality of diabetes-specific care received by older adults with diabetes in Qazvin, Iran. The study found that the mean scores on the PACIC-5As and its domains were higher than 3.0, which is similar to scores found in diabetes patients in the USA [22], Germany [48], and Switzerland [49]. This suggests that older Iranian patients receive a reasonable level of patient-centered care in accordance with the CCM.

The level of education was found to be a significant predictor of older patients’ perspective on diabetic care. Patients with higher education levels had higher PACIC-5As scores. Likewise, Rajabpour et al. (2019) [50] reported that patients with higher education had a greater perception of holistic care. Zakeri-moghadam and Sadeghi (2013) [51] also discovered a direct and significant relationship between patients’ education levels and their satisfaction with nursing services. This result may be attributed to the higher expectations of less educated patients from the healthcare staff and their care, and their lesser adaptation to their existing condition.

The CFA results confirmed the presence of five factors, which are assess, advise, agree, assist, and arrange, for the 25 items of the PACIC-5As questionnaire. Glasgow et al. (2005), the developers of the questionnaire, also confirmed the presence of these five factors based on the CMC model for patients with one or multiple chronic diseases [22]. Similarly, Wensing et al. (2008) [25] confirmed the existence of these five factors in Dutch patients with diabetes or chronic obstructive pulmonary disease (COPD) for the PACIC or PACIC-5 A questionnaire. Additionally, Shah et al. (2008) [37] verified the same for diabetic patients, and Rosemann et al. (2007) [20] for patients with osteoarthritis. However, Drewes et al. (2012) [52] could not replicate the five-factor structure in diabetic patients and long-term care patients. Similarly, Schwenke et al. (2019) [36] did not find the five-factor structure for the PACIC-5 A questionnaire among obese patients. These contradictory results may be due to methodological differences and the specific characteristics of the study samples. The statistical methods used in the studies were quite different, and those that conducted the EFA used various methods to determine the number of factors. For instance, Drewes et al. (2012) [52] focused on patients with multiple chronic conditions and employed both EFA and CFA using the split-half method. They used three types of EFA (Principal axis factoring, PAF) with oblimin rotation, parallel analysis (PA), and Velicer’s minimum average partial (MAP) test] to explore the factor structure of the data. On the other hand, Schwenke et al. (2019) [36] examined the perspective of obese patients and used EFA with PA and the eigenvalue criterion and scree plot.

The reliability of the instrument in our study, as measured by Cronbach’s alpha, was in the acceptable range (Cronbach’s alpha = 0.805). In a study by Glasgow et al. (2005) [22], a Cronbach’s alpha of 0.96 for PACIC and 0.97 for PACIC-5As was reported in diabetic patients. Schwenke et al. (2019) [36] also reported a Cronbach’s alpha of 0.93 for PACIC and 0.94 for PACIC-5As in obese patients. Additionally, Alharbi et al. (2016) [35] found a Cronbach’s alpha of 0.93 for PACIC and 0.90 for PACIC-5As. The findings from previous studies demonstrated an optimal level of internal consistency. The variation in Cronbach’s alpha values in these studies may be attributed to the differences in the sample being investigated.

### Limitations

Since this study was conducted on older patients with diabetes, the results may not be generalizable to older individuals with other chronic diseases. Another limitation is the response bias, including social desirability bias that seems relevant to the geriatric population. To mitigate this limitation, we provided necessary explanations regarding the study’s objectives and the application of the results to improve the care of patients with diabetes. Another limitation of the current research is the lack of test-retest reliability assessment due to limited access to the study’s older participants. It is recommended that future studies examine the temporal stability of the assessment tool.

## Conclusion

The results of this study indicated that the PACIC-5As is a reliable and valid tool for measuring quality of care based on the key components of the CCM and patient motivation using the “5 A” principles in older Iranian patients with diabetes. This tool can also evaluate how well physicians’ counseling reflects the 5 A-approach, which involves assessing, advising, agreeing, assisting, and arranging. Using this instrument can guide health professionals and policy-makers in Iran to improve the healthcare delivery system for older diabetic patients and enhance their satisfaction with the care they receive. In the present study, the 5As analysis showed that the scores obtained from “Arrange” and “Assiste” were less than the other “As” among older patients. So, it is suggested to use practical and cost-effective interventions for improving older patients’ perspective on diabetic care.

## Supplementary Information

Supplementary Material 1.

Supplementary Material 2.

## Data Availability

All data generated or analyzed during this study will be available from the corresponding author on reasonable request.

## Abbreviations

PACIC-5As: Patient Assessment of Chronic Illness Care
WHO: World Health Organization
Chronic Care Model: CCM
US: United State
CVR: Content Validity Ratio
CVI: Content Validity Index
CFA: Confirmatory Factor Analysis
SEM: Structural Equation Modeling
VIF: Variance Inflation Factor
SD: Standard Deviation
IQR: Interquartile Range
RMSEA: Root Mean Square Error of Approximation
GFI: Goodness of Fit
AGFI: Adjusted Goodness of Fit Index
IFI: Incremental Fit Index
CFI: Comparative Fit Index
SRMR: Standardized Root Mean Square Residual
