# Supplementary material for: Transcultural adaptation and validation of Persian Version of Patient Assessment of Chronic Illness Care (PACIC-5As) Questionnaire in Iranian older patients with type 2 diabetes
Source: BMC Health Serv Res. 2024 Sep 16;24:1073. doi: 10.1186/s12913-024-11557-0 (PMC11404006; doi:10.1186/s12913-024-11557-0)
Supplement: Supplementary file 1 — Supplementary Material 1. [file 12913_2024_11557_MOESM1_ESM.docx]

**Patient Assessment of Chronic Illness Care (PACIC-5As) Questionnaire**

| **Always** | **Most of the times** | **sometimes** | **rarely** | **Never** | **item** |  |
| --- | --- | --- | --- | --- | --- | --- |
|  |  |  |  |  | Asked for my ideas when we made a treatment plan | 1 |
|  |  |  |  |  | Given choices about treatment to think about | 2 |
|  |  |  |  |  | Asked to talk about any problems with my medicines or their effects | 3 |
|  |  |  |  |  | Given a written list of things I should do to improve my health | 4 |
|  |  |  |  |  | Satisfied that my care was well organized | 5 |
|  |  |  |  |  | Shown how what I did to take care of my illness influenced my condition | 6 |
|  |  |  |  |  | Asked to talk about my goals in caring for my illness | 7 |
|  |  |  |  |  | Helped to set specific goals to improve my eating or exercise | 8 |
|  |  |  |  |  | Given a copy of my treatment plan | 9 |
|  |  |  |  |  | Encouraged to go to a specific group or class to help me cope with my chronic illness | 10 |
|  |  |  |  |  | Asked questions, either directly or on a survey, about my health habits | 11 |
|  |  |  |  |  | Sure that my doctor or nurse thought about my values and my traditions when they recommended treatments to me | 12 |
|  |  |  |  |  | Helped to make a treatment plan that I could carry out in my daily life | 13 |
|  |  |  |  |  | Helped to plan ahead so I could take care of my illness, even in hard times | 14 |
|  |  |  |  |  | Asked how my chronic illness affected my life | 15 |
|  |  |  |  |  | Contacted after a visit to see how things were going | 16 |
|  |  |  |  |  | Encouraged to attend programs in the community that could help me | 17 |
|  |  |  |  |  | Referred to a dietitian, health educator or counsellor | 18 |
|  |  |  |  |  | Told how my visits with other types of doctors, an eye doctor or surgeon, helped my treatment | 19 |
|  |  |  |  |  | Asked how my visits with other doctors were going | 20 |
|  |  |  |  |  | Asked what I would like to discuss about my illness at that visit | 21 |
|  |  |  |  |  | Asked how my work, family or social situation related to taking care of my illness | 22 |
|  |  |  |  |  | Helped to make plans for how to get support from my friends, family or the community | 23 |
|  |  |  |  |  | Told how important the things I do to take care of my illness (e.g. exercise) were for my health | 24 |
|  |  |  |  |  | Set a goal together with my team for what I could do to manage my condition | 25 |
|  |  |  |  |  | Given a book or monitoring log in which to record the progress I make | 26 |
