# Supplementary material for: Transcultural adaptation and validation of Persian Version of Patient Assessment of Chronic Illness Care (PACIC-5As) Questionnaire in Iranian older patients with type 2 diabetes
Source: BMC Health Serv Res. 2024 Sep 16;24:1073. doi: 10.1186/s12913-024-11557-0 (PMC11404006; doi:10.1186/s12913-024-11557-0)
Supplement: Supplementary file 2 — Supplementary Material 2. [file 12913_2024_11557_MOESM2_ESM.docx]

**Persian version of PACIC-5As questionnaire**

پرسشنامه ارزیابی بیمار از مراقبت بیماری مزمن (PACIC-5As)

|  | **آیتم ها** | هرگز | به ندرت | گاهی | بیشتر اوقات | همیشه |
| --- | --- | --- | --- | --- | --- | --- |
| 1 | در زمان تدوین و تنظیم برنامه درمانی، با من مشورت می کردند. |  |  |  |  |  |
| 2 | روشهای درمانی را معرفی میکردند تا در موردشان فکر کنم. |  |  |  |  |  |
| 3 | از من میخواستند درباره مشکلاتی که با داروها یا اثرشان دارم، صحبت کنم. |  |  |  |  |  |
| 4 | لیست کارهایی که باید در جهت بهبود وضعیت سلامتیم انجام دهم را به من ارائه می دادند. |  |  |  |  |  |
| 5 | از نحوه سازماندهی مراقبت های ارایه شده، رضایت داشتم. |  |  |  |  |  |
| 6 | به من نشان میدادند چگونه آنچه در جهت مراقبت از خود انجام میدهم بر وضعیت بهبودیم، تاثیر میگذارد. |  |  |  |  |  |
| 7 | از من میخواستند، اهداف مراقبت از بیماریم را بیان کنم. |  |  |  |  |  |
| 8 | به من کمک میکردند تا اهداف مشخصی برای بهتر شدن ورزش و تغذیه خود مشخص کنم. |  |  |  |  |  |
| 9 | نسخه ای از برنامه درمانی را به من تحویل می دادند. |  |  |  |  |  |
| 10 | تشویقم میکردند تا به یک گروه یا کلاس خاص بپیوندم تا بتوانم با بیماریم کنار بیایم. |  |  |  |  |  |
| 11 | درباره عادات سلامتیم، چه بطور مستقیم و چه در نظرسنجی، پرس وجو میکردند. |  |  |  |  |  |
| 12 | مطمئنم که پزشک یا پرستارم در زمان تنظیم برنامه درمانیم، ارزش ها و باورهای فرهنگیم را در نظر می گرفتند. |  |  |  |  |  |
| 13 | کمکم میکردند که برنامه درمانی تنظیم کنم که در زندگی روزانه ام، قابل اجرا باشد. |  |  |  |  |  |
| 14 | کمکم میکردند تا برنامه درمانیم را پیش ببرم و بتوانم حتی در موقعیتهای سخت و دشوار، دست از درمان نکشم. |  |  |  |  |  |
| 15 | از من میپرسیدند که بیماریم چه تاثیری بر زندگی شخصیم گذاشته است. |  |  |  |  |  |
| 16 | پس از ویزیت با من تماس گرفته و وضعیت سلامتیم را پی گیری می کردند. |  |  |  |  |  |
| 17 | تشویقم میکردند که در برنامه های اجتماعی موثر بر بهبود سلامتیم، شرکت کنم. |  |  |  |  |  |
| 18 | من را به یک متخصص تغذیه، مشاور و یا کارشناس سلامت ارجاع میدادند. |  |  |  |  |  |
| 19 | برای من توضیح میدادند که ویزیت سایر پزشکها (چشم پزشک و جراح) چه کمکی به درمانم میکند. |  |  |  |  |  |
| 20 | در مورد نحوه انجام ویزیت سایر پزشکان، از من سوال می کردند. |  |  |  |  |  |
| 21 | از من، در مورد مشکلات مربوط به بیماریم که تمایل دارم با پزشکان دیگر صحبت کنم، سوال می کردند. |  |  |  |  |  |
| 22 | درباره تاثیر شغل، خانواده و وضعیت های اجتماعی بر برنامه درمانیم، از من میپرسیدند. |  |  |  |  |  |
| 23 | در برنامه ریزی برای دریافت حمایت از سوی خانواده، دوستان و جامعه به من کمک می کردند. |  |  |  |  |  |
| 24 | اهمیت کارهایی که برای بهبود بیماریم انجام میدهم (مثل ورزش) را برای من توضیح میدادند. |  |  |  |  |  |
| 25 | اهداف مورد انتظار برای مدیریت بیماری خود را با کمک تیم درمان، تعیین می کردم. |  |  |  |  |  |
| 26 | کتابچه نظارت بر درمان جهت ثبت و ارزیابی پیشرفت درمانم، به من دادند. |  |  |  |  |  |
